# Scoping review of international relations theories in health security: A cue for health diplomacy

**DOI:** 10.12688/f1000research.145568.1

**Published:** 2024-03-11

**Authors:** Sanjay Pattanshetty, Aniruddha Inamdar, Viola Savy Dsouza, Kiran Bhatt, Amrita Jash, Nachiket Gudi, Helmut Brand

**Affiliations:** 1 Centre for Health Diplomacy, Department of Global Health Governance, Prasanna School of Public Health, Manipal Academy of Higher Education, Manipal 576104, Karnataka, India; 2 Department of International Health, Care and Public Health Research Institute—CAPHRI, Faculty of Health, Medicine and Life Sciences, Maastricht University, 6211 LK Maastricht, The Netherlands; 3 Centre for Regulatory Science, Department of Health Information, Prasanna School of Public Health, Manipal Academy of Higher Education, Manipal, Karnataka, 576104, India; 4 Department of Geopolitics and International Relations, Manipal Academy of Higher Education, Manipal, Karnataka, 576104, India; 5 Department of Health Information, Prasanna School of Public Health, Manipal Academy of Higher Education, Manipal, Karnataka, 576104, India; 6 Department of Health Policy, Prasanna School of Public Health, Manipal Academy of Higher Education, Manipal, Karnataka, 576104, India

**Keywords:** Health security, international relations theories, realism, liberalism, securitization

## Abstract

**Background:**

Health security as a domain has gained tremendous importance in the recent past. Emerging and re-emerging diseases globally, coupled with the derailment of the determinants of health mainly the socio-political environment, has made health security a cross-cutting entity in diverse fields including International Relations (IR). With the ongoing global polycrisis, the health-related issues which were previously sidelined as a concept of less strategic importance in the IR field, are now contributing to the shift of the world order. This has instilled an increased participation of IR scholars in the discussions and debates on health security concerns. The field of IR contains numerous theoretical lenses through which scholars analyze such situations, policies, and systems of the world.

**Methods:**

In this paper, we use a scoping review method to inspect how IR theories have been applied in analyzing health security concerns.

**Results:**

We observed that various diverging IR theories have been used to deliberate on states’ actions in tackling the recent pandemic and have also been prescriptive about the changing notions of multilateralism and international governing organizations. Realism, liberalism, and securitization were among the most frequently applied IR theories in the context of health security discussions.

**Conclusions:**

This work provides an impetus to enhance the interaction among interdisciplinary teams leading to evolving solutions that can address issues of global importance in the contemporary world.

## Introduction

The COVID-19 pandemic has compelled us to reimagine the existing world order and inspect the nature of the shift that can be expected. ^1^^–^^3^ With health security becoming the forefront of the discussion in national security and foreign policy concerns, governments worldwide had to balance their act of protecting their people while having a global outlook. This situation was further aggravated by tensions between the USA and China on the origin of the COVID-19 virus, China’s contention over Taiwan’s accession to the World Health Organization (WHO), and the Russia-Ukraine conflict. There is also a resounding agreement among the countries on the reformation of multilateral systems due to the challenges it brought in mitigating the health and geopolitical crises. ^4^ These situations have ignited discussions and debates among International Relations (IR) scholars who have tried to analyze the world’s situations, policies, and systems. ^5^

IR scholars generally use the theoretical knowledge base of the field to understand and elaborate on how countries respond to changes and challenges at the global level and navigate inter-state dynamics. Theories are instruments designed to help understand a particular phenomenon that function as a guide for research to explain a specific event or predict the occurrence of something. ^6^ These theories are also utilized by political leaders and policymakers in articulating solutions at the national and international levels which later translate into policies framed to obtain national objectives, which vary in their strategic importance. While states have been the primary focus of IR, the role played by other actors, such as international organizations, civil society groups, and even individuals, has gained prominence in the discourse. However, in the pursuit of peace and security, IR as a field has broadened its horizon by interacting with dynamic fields like global health to analyze how the context of conflicts, development inequity, socio-economic conditions, environmental volatility, and global governance affect health security. ^7^

WHO defines global public health security “as the activities required, both proactive and reactive, to minimize the danger and impact of acute public health events that endanger people’s health across geographical regions and international boundaries.” The pandemic magnified national conflicts and challenges impacting the lives of billions globally. In many countries, the crisis turned into a political test. ^8^ The polycrisis nature of the pandemic has created tensions in international relations and health security. ^9^ Historically, health issues largely remained outside the purview of most traditional IR scholarly works as it was treated with lesser strategic significance. However, the importance of discussing health security in the IR field was recognized during the post-Cold War period with the emergence of new security threats, particularly the prospects of pandemics related to emerging/re-remerging diseases and the increased perils of bioterrorism. This led to the issue of health security being dealt with under the ambit of foreign, national, and security policies. Ever since, the aspects of health security have been studied in IR concerning governance, inter-governmental institutions, globalization, diplomacy, and others.

While health-related debates have gradually made their way into IR scholarly works, there is a need for deeper engagement among IR scholars on health. The changing context of the health and foreign policy interface has emphasized health security as a valid concern for IR and could find a place within the traditional boundaries of IR theories. Thus, this scoping review intends to understand how IR scholars have debated and discussed health concerns through a theoretical lens to gauge its potential impact on the international world order.

## Methods

A scoping review approach enables us to understand emerging evidence and is regarded as a first step to research evidence development. ^10^ We have followed the framework espoused by Arskey and O’Malley (2005) to inform the methods of this review. ^11^ The PRISMA-ScR (Preferred Reporting Items for Systematic Reviews and Meta-Analyses Extension for Scoping Reviews) checklist was followed to report the review. ^11^^,^^12^

### Step 1: Identifying the research question

The research question was identified by SP in consultation with the subject matter expert (HB) and further refined with the consensus of the research team. Since this is an emerging domain, we inquired about the following research question:

“How have International Relations scholars discussed and debated on health security concerns”?

Since our objective is to understand the discussions on how these IR scholars have discussed health issues and that the discussion would be informed by a theoretical underpinning in the health security domain, we have adapted the health policy triangle framework instead of other frameworks. ^13^^,^^14^

### Step 2: Identifying relevant studies

Keyword searches were conducted in the English language in PubMed (NCBI), Web of Science (Elsevier), Scopus (Elsevier), and Embase (Elsevier) databases on November 5 ^th^, 2023, to cover a range of literature using the following search terms: “international relations theories” AND “health security”. The search strategy used for the study is provided in Appendix 1 of the Extended data. ^15^ We further performed a search on Google Scholar with the aforementioned terms and screened the first five pages (with 10 entries in a page) for possible inclusion. The more detailed search strategy is in an online supplementary document. The search terms were based on the focus of the various IR theories and commonly used terms related to health security. Searches were limited to peer-reviewed journal articles they were not limited to any period. All the records were exported to Rayyan.Ai, a reference management software, and deduplication was carried out. ^16^

Step 3: Study Selection

The study selection was carried out at two stages, namely Title-abstract (Ti-Ab) and full-text stages. Ti-Ab screening was conducted by SP, KB, NG, and AI independently while screening at the full-text stage was conducted by SP, AI, and KB based on the following selection criteria: The inclusion criteria comprised of two aspects: (1) the articles needed to reference international relations theories explicitly, and (2) they should extensively apply these theories to offer valuable insights into understanding positions, negotiations, and outcomes in health security. In disagreements, a resolution was sought through discussion and consensus involving a third review author (VD).

### Steps 4 and 5: Charting the data and collating, summarising, and reporting the results

Three review authors independently extracted information from the articles, utilizing a pre-piloted electronic data extraction form. When discrepancies arose, the two review authors engaged in discussions to reach a consensus. The third author participated in further deliberations if a resolution could not be reached. The records at each review stage are presented in the PRISMA flow diagram. The data synthesis process incorporated principles derived from Walt and Gilson’s Health Policy Triangle framework model. ^14^ The framework was employed because it offers a perspective that allows for the analysis of the interactions between actors, context, content, and processes. This framework facilitates an insightful approach to understanding the application and influence of different International Relations (IR) theories in the realm of health security. In this structure, the columns were categorized into distinct components such as “condition” “context” and “content”. The gathered data is then synthesized for a theme, along with a comprehensive analysis of the supporting evidence within each theme.

## Results

A total of 12,889 records were retrieved from a database search Web of Science (Clarivate) – 2802, PubMed (NBCI) – 1362, Scopus (Elsevier) -6275, and Embase (Elsevier)- 2560. We removed 5227 duplicates from the list of articles. After the screening, 10 studies that met the eligibility criteria were included in the synthesis. We screened 50 records from Google Scholar (from the first five pages) and included 14 records for the synthesis. The PRISMA flow chart which is available as part of extended data, depicts the detailed study selection. ^15^ Characteristics of the included studies and the arguments made by the authors are presented in Appendix 2 and Appendix 3 of the extended data. ^15^

The data synthesis table involved extracting the IR theories discussed during the discussion of health security, health conditions presented in the paper, the context discussed, and the contents of the application of the theory. The detailed data synthesis table is provided in Table 1. The selected studies showed that a few papers used multiple theories in a single study and often presented arguments from various points of view to make a case. The theory of realism, securitization, and liberalism were the most frequently applied theories discussing health security.

**Table 1.** Data synthesis.

Theory	Condition	Context - Keywords	Content - Summary
Realism ^1^^–^^3^^,^^5^^,^^17^^–^^19^	COVID-19	- International cooperation and liberalism have fallen into a crisis - Lack of global leadership - Opposing political ideologies - Vaccine nationalism	Lack of sufficient global health cooperation due to conflicts and power interests reflected in the international governance structure. Vaccine nationalism enabled states to act for their self-interests and power while cooperating with others.
Liberalism ^1^^,^^3^^,^^5^^,^^18^^,^^19^	COVID-19	- United States-led liberal international order - Emergence of a post-liberal international order - International cooperation - Middle-power countries in the East Asian region	The main components of liberal theory are interdependence, transnationalization, growth of international institutions, and democracy. Unparalleled research cooperation emerged during COVID-19-19, where the standard protocols were bypassed. Strengthening international cooperation and avoiding nationalism is critical to addressing the global health crisis.
Securitisation ^13^^,^^17^^,^^20^^–^^31^	HIV, Pandemic influenza, SARS, H5N1, H1N1, lifestyle diseases and mental health, Pandemic, Tobacco, Infant deaths, diarrheal diseases, COVID-19	- Need for emergency policies - Neoliberal governments to roll out unpopular economic reforms - The securitization lens is prominently used in analyzing Global Health Security (GHS) - Difficulty of de-securitizing	Securitization highlights the need to prioritize and address unconventional threats. The statist approach (securitization) demands health initiatives to be associated with foreign or defense policy concerns. The responses of securitization of health shift the efforts considered being altruistic to one of self-interest. The ahistoricism of securitization theory and its assumptions of social and international security are analytically divisible and have to be challenged.
Constructivism ^5^^,^^19^^,^^32^	food, housing, clothes, minimum education, and some health services, COVID-19	- Meanings, purposes, and objectives associated with technological development. - Understanding global and national policy responses to the crisis - To examine the problem of why states fail to cooperate	The reality is inter-subjective and not objective as Realist and Liberals propose. The constructivist approach stresses the importance of belief structures, identities, and roles. A consensus on reality is necessary among actors in international politics to provide appropriate responses.
Critical Security Theory ^13^	Global Health	- To investigate the intertwining nature of national health policies and provisions with international considerations.	Well-being and individual rights are paramount for the globalist approach (critical security theory) that seeks the advancement of health policies.
Critical Theory ^3^	COVID-19	- Investigation of the geostrategic developments	Critical theorists emphasize that unjust economic and ideological structures of the existing world order have led to the mismanagement of the recent pandemic. The 'cost-benefit' reason based on 'instrumental reason' for policy actions is criticized. A 'benefit-benefit' logic based on 'moral-transcendental rationality' is promoted.
Decolonization Theory ^24^	COVID-19	- Understanding and analysis of global health diplomacy from a relational view of power	Decolonization theory was manifested in the inequity in COVID-19 vaccine distribution.
Liberal institutionalism ^18^	COVID-19	- Security-based international order to establish global justice	Liberal Institutionalists contend that international order is affected by domestic political regimes. International order derives from the power of international norms, laws, and institutions.
Marxist Theory ^19^	Health and Social Justice	- Identifying the drivers of global health diplomacy	Marxist theory's sub-imperialism discussions are essential to understanding the foreign relations of Southern countries in the context of global capitalism.
Modern Socialist ^19^	Health and Social Justice	- To theorize (and practice) anti-hegemonic alternatives to mainstream international relations	An egalitarian exchange in international relations fuelled by the collective action of the oppressed is envisioned by modern socialists.
Neo-Marxist Theory ^33^	Health inequalities	- Examining the key concepts and ideas within Neo-Marxist class analysis - Utilized to analyze and explain health disparities.	Neo-Marxist studies reveal a genuine lack of correspondence between theoretical definitions and social class measures.
Normative Theory ^5^	COVID-19	- Grasping global and national policy responses to the crisis - To understand the problem of why states fail to cooperate in the pursuit of common interests	The normative theory centers on a moral judgment about what should be. The post facto character of the theory is not able to explain to analysts the patterns and functionality of international cooperation.
Post-Modernist ^34^	Communicable and NCD	- The etiological multifactorial character of the elements involved in disease pathogens - New protagonists are manifesting at the international level, The interest in state problems gets diminished	Health protection may be thematically integrated within the concept of security. The extreme postmodernist vision is when states are pushed towards the removal of economic barriers to trade in the context of the globalization of health where interests of communities, international organizations, corporations, and others become the drivers.
Theory of Hegemonic Rise and Fall ^18^	COVID-19	- Use of the proposed conceptual model to predict the future international order	The cycle of rise and fall of great powers occurs due to the law of uneven growth causes and the strength of international political currency of economy and military.
Theory of Relational Justice ^17^	Health Diplomacy	- Positioning global health better within foreign policy	The theory of relational justice states that there are no simply ‘positive duties’ to assist, but moral obligations to prevent harm.

The increased relevance of discussions on international relations and health security during the recent COVID-19 pandemic was reflected in our scoping review with a majority of articles focusing on it. These papers primarily deliberated on how states across the world handled the pandemic and how we can anticipate a shift in the existing world order using the existing IR theories. However, one theory that stood out with its application in various global health challenges is the theory of securitization. This theory has been not only applied during COVID-19 but also in HIV, Pandemic influenza, SARS, H5N1, H1N1, infant deaths, diarrheal diseases, tobacco, ‘lifestyle diseases’, and ‘mental health’. The prominent global health concern of non-communicable diseases (NCDs) was discussed in a paper that discussed the role of Post-modernist IR theory in health security. The IR discussions have also taken place on determinants of health such as food, housing, and education through the lens of constructivism. Additionally, social justice and health inequalities concerns have been dealt with by IR scholars. Thus, IR theories have tried to emphasize the relevance of health security’s geopolitical and geoeconomic determinants.

The data on the context and content built by a particular IR theory is imperative to understanding the divergence and similarities in viewing health security. The papers that highlight the realist theory emphasize that the liberal world order that promotes global cooperation has failed during the COVID-19 crisis. They view that nations’ self-centered and self-interest-promoting actions in mitigating the pandemic’s risks and the rise of vaccine nationalism were the primary reasons for the lack of practical global cooperation and leadership. They foresee a shift in the US-led liberal world order that has been prevalent after the end of the Cold War.

In contrast to the realists, there is a greater emphasis on the liberal approach as being more relevant to the challenges of COVID-19. Liberals recognized the current world order’s limitations and highlighted the need for new geopolitical and geoeconomic players such as the middle-power countries in the East Asian region to enhance cooperation and fill the global leadership void. Despite the national interests becoming prominent in mitigating the pandemic, there has been an unprecedented rise in effective scientific collaborations for addressing the global health challenge. Thus, the liberals emphasize that strengthening cooperation and defying nationalistic measures is vital to tackling future global health crises.

‘Securitization theory’ has been the most recurring theory of IR that has contributed to the discussions of health security with its application across various global health concerns. The studies using this theory have stressed the importance of the securitization of health, which places ‘health’ as a foreign and defense policy domain and prioritizes its deliberation and action towards mitigating global health concerns. By making health a foreign policy priority, it challenges the liberal world order with an augmented tendency of states acting in self-interests above a global vision (statist approach). However, it is important to examine whether the securitization of health indeed translates to ‘health security’. The analytical demarcation of social and international security of the securitization theory must be addressed as the prolonged securitizing of health threats’ has become a concern for efficient resource re-allocation.

The studies that used constructivism theory have used it to understand the reasons for the lack of effective cooperation despite common interests among nations. It emphasizes that an interlinkage between global and national policies is imperative to address health crises. It diverges from the realist and liberal views of the objectivity of states’ actions and highlights its subjective and complex nature. According to the theory, the consensus of the reality in which states function affects the state’s responses to a common concern and interest.

The critical security theory has been utilized to highlight the need for a globalist approach in which health policies focus on well-being and individual rights. The application of critical and decolonization theory was made to understand the inequitable nature of the world in terms of economic and political power and its effects on the management of the pandemic. With the rise of scrutiny on international organizations and the shift in the world order, liberal institutionalists who examine their determinants have emphasized the importance of domestic regimes and international norms for collective action against global health challenges. Modern Socialist and Marxist Theories were highlighted in a study that endorsed South-South cooperation in health through the lens of social justice. The Neo-Marxist theory, predominantly used to explain health inequity, was also used to explain the divergence in understanding social class.

Health security has also been looked at through the normative theory, which is prescriptive on the existence of effective international agreements and cooperation. However, the study that discusses it also highlighted its limitation of inability to explain the crumbling nature of international cooperation that deterred a comprehensive mechanism to address health challenges. While liberals argued for new developing state actors to cooperate for collective action, the post-modernists argued that such cooperation must also involve the voice of all stakeholders. The discussion of new actors becomes prevalent when the discussions by the theory of hegemonic rise and fall highlight the cyclical nature of great powers and the lack of global leadership in mitigating health risks. This brings importance to why health has to become a foreign policy priority in all countries as deliberated by the theory of relational justice which emphasizes that states should not only assist in treating a health concern that affects them but also in preventing any future harm to other countries.

## Discussions

The global health challenge of the COVID-19 pandemic has invariably threatened the primary functions of foreign policy, such as international security, economic stability, human dignity, and social justice. The lapse of the existing global health systems, governing structures, and geopolitical tensions across the world, have made it relevant for international relations scholars to discuss health security and the future direction of the global world order. In this scoping review, we witnessed increased instances of applying IR theories in health security discussions to explain the complex nature of the shifting world order. Various diverging theories have been used to deliberate on states’ actions in tackling the recent pandemic and have also been prescriptive about the changing notions of multilateralism and international governing organizations. Additionally, the IR theories have also discussed other health concerns such as communicable and non-communicable diseases, and the determinants of health such as food, housing, education, social justice, and inequalities. Thus, it has become more imperative than ever before to understand how health is evolving into a critical concern in IR.

The contentious issues with the geopolitical dimension are mainly linked to equity and human rights, the sharing of knowledge, technology, and innovation, and the financing of global public goods for health. ^8^ The lack of global solidarity is the major barrier to achieving equity in access to vaccines, therapeutics, and diagnostics. According to WHO Director-General, Dr Tedros Adhanom Ghebreyesus, from the human rights lens, “[t] he enjoyment of the highest attainable standard of health is one of the fundamental rights of every human being without distinction of race, religion, political belief, economic or social condition”. The central principle of the 2030 Agenda for Sustainable Development is to ensure that no one is left behind. For instance, inequity and lack of international cooperation to access COVID-19 tools threatened global public health security.

The involvement of IR theories in discussions on health has evolved post-Cold War caused by the shift in the lens through which policymakers view health. Security discussions during the Cold War period focused predominantly on military-based national security concerns. According to the WHO Regional Committee for the Eastern Mediterranean, the security dimensions in the 21 ^st^ century are characterized by increasing complexity, the fourth industrial revolution, economic polarization, global interdependence in the supply chain, international conflicts, and a wide range of threats, including emerging and re-emerging diseases. The growing insecurity is not adequately addressed by the traditional mechanisms used to pursue national security. In the current scenario, people’s security depends on factors beyond a particular state’s control and demands a global approach to tackle fundamental issues and mutual vulnerability. The complexity of the global problems and the need for collective action have made it relevant for a multidisciplinary approach to tackle global concerns.

The increased securitization of global public goods and services, such as health, has provided the impetus for IR scholars to contribute to its discussions. The gradual shift of health from being of ‘low politics’ (humanitarian endeavor) to ‘high politics’ (national and international security concerns) and its increased discussions in foreign policy matters have provided a perfect segway for applying IR theories in such conditions. ^35^ The results of this study show that the IR theories deliberating on health concerns have disease-specific and system-oriented elements. The theories have been applied to understand the state’s action or inaction during a health crisis and relate it to the geopolitical environment in which they function. Interestingly, despite their differences in their approach to tackling a crisis, most theoretical applications concur that the world can expect a shift in the world order after the COVID-19 pandemic. Thus, by looking at geopolitical and geoeconomic determinants of health, the theories have emphasized the need for health to be considered a foreign policy issue.

This scoping review intended to understand how IR theories were applied in health security, why it is relevant in the current geopolitical and health context, and what difference they can make. The results have extensively shown the overlaps and the variance in the theoretical approach to health security. While the wake-up call for IR scholars to deliberate on health security issues became prominent with the COVID-19 pandemic, health as a non-traditional security issue evolved in the post-Cold War era to be included and prioritized in foreign policy. The momentum that health security has gained in the IR space must be further pushed forward by IR scholars using the existing theories in post facto analysis and predicting and prescribing states’ actions. Thus, by combining the two disciplines of IR and health, the world can be prepared to tackle a future global health crisis that implies geopolitics. We included studies published only in the English language and we acknowledge this as a limitation. Future studies can include literature from other languages.

## Conclusions

This scoping review brings into perspective the need for adopting an interdisciplinary as well as a multidisciplinary approach in bridging the gap between health and international relations. The COVID-19 pandemic brought to the surface that health crisis is not just a single state’s problem and demands a global approach to finding solutions to such crisis. Pandemics thereby are a ‘global health issue’ and very much linked to states ‘security’ and ‘survivability’. This makes it imperative to view and discuss ‘health’ in the larger confines of IR. Given this, the paper attempts to further expand the security discourse of IR by focusing on ‘health’ as a core issue of security. Here, the intention lies in creating the scope for further research to bridge the existing gap between ‘health’ and ‘IR’.

## Data Availability

All data underlying the results are available as part of the article and no additional source data are required. Open Science Framework: Scoping Review of International Relations Theories in Health Security: A Cue for Health Diplomacy. https://doi.org/10.17605/OSF.IO/NY9WZ. ^15^ This project contains the following underlying data:
• Appendices.pdf (Appendix 1 - Literature Search, Appendix 2 - Reason for excluding studies, Appendix 3 - Data Extraction Table) • PRISMA Flowchart.jpg PRISMA-ScR checklist for ‘Scoping Review of International Relations Theories in Health Security: A Cue for Health Diplomacy’. https://doi.org/10.17605/OSF.IO/NY9WZ. ^15^ Data are available under the terms of the Creative Commons Zero “No rights reserved” data waiver (CC0 1.0 Public domain dedication).
