# Supplementary material for: Scoping review of international relations theories in health security: A cue for health diplomacy
Source: F1000Res. 2024 Mar 11;13:184. [Version 1] doi: 10.12688/f1000research.145568.1 (PMC11109696; doi:10.12688/f1000research.145568.1)
Supplement: Peer review history

# Reviewer report: Reviewer response for version 1

**DOI:** 10.5256/f1000research.159537.r263569

**Published:** 2024-05-21

**Authors:** Garry Aslanyan

**Affiliations:** 1 Dalla Lana School Of Public Health, University of Toronto, Toronto, Ontario, Canada

As a scoping review, this article provides useful overview of theories used in IR and it is applied in analyzing health security. The paper can be further strengthened by clearly outlining which stakeholders in IR (academia, think tanks, governments, lobby groups, NGOs) need to do what differently to adjust to the realities of 21st century and the need for a multidisciplinary approaches in these theories going forward.

Are the rationale for, and objectives of, the Systematic Review clearly stated?

Yes

Is the statistical analysis and its interpretation appropriate?

Not applicable

If this is a Living Systematic Review, is the ‘living’ method appropriate and is the search schedule clearly defined and justified? (‘Living Systematic Review’ or a variation of this term should be included in the title.)

Not applicable

Are sufficient details of the methods and analysis provided to allow replication by others?

Partly

Are the conclusions drawn adequately supported by the results presented in the review?

Partly

Reviewer Expertise:

health policy and systems, global health diplomacy

I confirm that I have read this submission and believe that I have an appropriate level of expertise to confirm that it is of an acceptable scientific standard.

# Reviewer report: Reviewer response for version 1

**DOI:** 10.5256/f1000research.159537.r263573

**Published:** 2024-05-09

**Authors:** Anders Nordström

**Affiliations:** 1 Karolinska Institutet, Stockholm, Sweden

-The article is focused with a clear question as well as results.

 I have only three comments

 1. The fact that health security has been interpreted in different ways in IR - what does that mean? Any implications? Recommendations for  the future?

 2. The article indicates that health treated as a security issue is (or was?) now higher up on the political agenda. It would be useful with some comments on the fact that that interest has now disappeared i.e. lack of sustainable political interest

 3. Are the risks and limitations applying an IR perspective and analysis of health?

Are the rationale for, and objectives of, the Systematic Review clearly stated?

Yes

Is the statistical analysis and its interpretation appropriate?

Yes

If this is a Living Systematic Review, is the ‘living’ method appropriate and is the search schedule clearly defined and justified? (‘Living Systematic Review’ or a variation of this term should be included in the title.)

No

Are sufficient details of the methods and analysis provided to allow replication by others?

Yes

Are the conclusions drawn adequately supported by the results presented in the review?

Yes

Reviewer Expertise:

Public health policy

I confirm that I have read this submission and believe that I have an appropriate level of expertise to confirm that it is of an acceptable scientific standard.

# Reviewer report: Reviewer response for version 1

**DOI:** 10.5256/f1000research.159537.r263570

**Published:** 2024-04-10

**Authors:** Rahul Jindal

**Affiliations:** 1 Surgery and Global Health, Uniformed Services University of Health Sciences (Ringgold ID: 377811), Bethesda, Maryland, USA; 2 Indian Institute of Public Health, Gandhinagar, Gujarat, India

The authors have carried out a scoping review to dissect the question “How have International Relations scholars discussed and debated on health security concerns”?

 The review has been carried out appropriately by employing standard methods adapting the health policy triangle framework.

**A few minor points will enhance the manuscript:**

 1. The authors need to clarify if gray literature was examined.

 2. The authors could add a few lines on the increasing geo-political influence of India and its’ potential role of leadership of the Global South as it relates to international relations and health security.

 3. The authors could also discuss the role of South-South collaboration in the new international world order.

 4. The authors have touched on ‘Modern Socialist and Marxist Theories’ and how this could be reconciled with universal human rights, a topic of great interest in liberal democracies.

 5. The authors briefly mention “The application of critical and decolonization theory” – they could perhaps expand on how decolonization of global health intersects with security concerns?

 Overall, this scoping review brings a new perspective in bridging the gap between health security and international relations. It is clearly important to understand international relations, security, global health, and diplomacy via a theoretical lens.

Are the rationale for, and objectives of, the Systematic Review clearly stated?

Yes

Is the statistical analysis and its interpretation appropriate?

Yes

Are sufficient details of the methods and analysis provided to allow replication by others?

Yes

Are the conclusions drawn adequately supported by the results presented in the review?

Yes

Reviewer Expertise:

Transplant surgery, Global Surgery, Global Health Diplomacy and development.

I confirm that I have read this submission and believe that I have an appropriate level of expertise to confirm that it is of an acceptable scientific standard.
